# EMT-Inducing Molecular Factors in Gynecological Cancers

**DOI:** 10.1155/2015/420891

**Published:** 2015-08-19

**Authors:** Loredana Campo, Catherine Zhang, Eun-Kyoung Breuer

**Affiliations:** ^1^Oncology Institute, Cardinal Bernardin Cancer Center, Loyola University Chicago, Maywood, IL 60153, USA; ^2^Department of Radiation Oncology, Stritch School of Medicine, Loyola University Chicago, Maywood, IL 60153, USA

## Abstract

Gynecologic cancers are the unregulated growth of neoplastic cells that arise in the cervix, ovaries, fallopian tubes, uterus, vagina, and vulva. Although gynecologic cancers are characterized by different signs and symptoms, studies have shown that they share common risk factors, such as smoking, obesity, age, exposure to certain chemicals, infection with human immunodeficiency virus (HIV), and infection with human papilloma virus (HPV). Despite recent advancements in the preventative, diagnostic, and therapeutic interventions for gynecologic cancers, many patients still die as a result of metastasis and recurrence. Since mounting evidence indicates that the epithelial-mesenchymal transition (EMT) process plays an essential role in metastatic relapse of cancer, understanding the molecular aberrations responsible for the EMT and its underlying signaling should be given high priority in order to reduce cancer morbidity and mortality.

## 1. Introduction

Epithelial-mesenchymal transition (EMT) is the conversion of epithelial cells to a mesenchymal phenotype, characterized by a loss of epithelial markers, such as E-cadherin, *β*-catenin, occludin, claudin, plakophilin, cytokeratin, and desmoplakins [1–3], and a gain of mesenchymal markers, such as N-cadherin, vimentin, and fibronectin [1, 3]. Studies have shown that dysregulation of certain transcription factors, oncogenes, tumor suppressors, miRNAs, and growth factor signaling can trigger the initiation of EMT, which is considered a major contributor to the poor prognosis in gynecological cancer.

Ovarian cancer is the most lethal gynecological cancer and tumor metastasis is responsible for its large disease mortality rate. The American Cancer Society (ACS) estimates 21,980 new cases of and 14,270 deaths from ovarian cancer in the USA in 2014 [4]. Ovarian cancer can start from epithelial cells, germ cells, and stromal cells. Platinum complexes and taxanes are usually the first treatment option given. However, about 20~30% of patients show resistance to the platinum-based chemotherapy [5, 6] and about 70~90% of patients who responded to the treatment experience a recurrence of cancer within a window of months to years [5, 7].

The ACS estimates 12,360 new cases of and 4,020 deaths from cervical cancer in the USA in 2014 [4]. Cervical cancer starts in a woman's cervix, which connects the lower part of the uterus to the vagina. HPV types 16, 18, and 31 cause most cervical cancers and vaccinations against HPV as well as the usage of condoms during sexual contact play a role in preventing HPV transmissions. The ACS recommends the administration of a Pap test every 3 years for women aged 21–29 and every 5 years for women aged 30–65 to detect malignancies and cervical intraepithelial neoplasias; early detection is key for good prognosis. While the incidence of cervical cancer has decreased in the past 40 years, it continues to be one of the most common gynecologic cancers in women [2].

Uterine cancer is commonly called endometrial cancer because it mostly starts in the endometrium, the inner lining of the uterus. The development of endometrial cancer is most prevalent in postmenopausal women. For populations within this category, it is highly recommended to have a pelvic exam every year and to report any vaginal bleeding as soon as possible to prevent the cancer metastasis. The ACS estimates 52,630 new cases of and 8,590 deaths from endometrial cancer in the USA in 2014 [4].

According to the ACS, about 4,850 new cases of and 1,030 deaths from vulvar cancer will occur in the USA in 2014 [4]. Cancer of the vulva is a rare disease. It forms in a woman's external genitalia and makes up 3 to 5% of all gynecologic malignancies [8]. Most vulvar cancers occur in older women after the development of precancerous variations called vulvar intraepithelial neoplasias (VIN) that last for several years before developing into cancer. It also can affect younger women infected with HPV types 16, 18, 33, and 35 [9].

Vaginal cancer is unusual and accounts for less than 2% of gynecologic malignancies with an expected 3170 new cases and 880 deaths in the United States in 2014 [4]. About 70% of cases of vaginal cancer are squamous cell carcinomas, which begin in the epithelial lining of the vagina. The main risk factors are HPV subtypes 16 and 18. In order to prevent this type of cancer, vaccination and the routine Pap test are highly recommended.

An increase in cellular survival, migration, invasion, metastasis, recurrence, and drug resistance is observed in gynecological cancers and may be due to EMT [2, 10–14]. Therefore, we strongly believe that investigating the dysregulation of molecular networks responsible for EMT and its consequences may be critical to a better understanding of the etiology of the cancers and development of new therapeutic modalities. In this review, we will discuss the current knowledge regarding factors involved in EMT in each of the gynecological cancers (Table 1).

## 2. Transcription Factors

Several studies have shown the importance of the Snail family of transcription factors in inducing EMT in cervical cancer, endometrial cancer, ovarian cancer, and vulvar cancer [1, 3, 9, 15]. The Snail family consists of zinc finger-containing transcription factors and includes Snail (SNAI1), Slug (SNAI2), and Smuc (SNAI3) [1]. In cervical cancer cells, Snail inhibits the expression of claudins, occludin, and thrombomodulin [1, 16, 17]. Snail and Smuc have both been associated with lymph node metastasis [15]. In endometrial cancer cells, Snail is overexpressed in both primary and metastatic tumors, and both Snail and Slug reduce E-cadherin expression [3]. Overexpression of Snail in ovarian cancer cells induced mesenchymal markers, such as tea-shirt zinc finger homeobox (TSHZ1), collagen type V*α*, and fibrillin-1 (FBN-1), while suppressing E-cadherin, myosin-5C, keratin-18, keratin-8, annexin-A3, and suppressor of tumorigenicity 14 (ST14) [18]. In vulvar cancer, both Snail and Slug inhibit the expression of E-cadherin and increase the expression of vimentin [9].

Zeb1/2 (collectively known as SIP1) are two-handed zinc factors that have been shown to cause EMT. In cervical cancer cells [1] and ovarian cancer cells [19], both transcription factors reduce the expression of E-cadherin. Zeb1 also induced the upregulation of vimentin in cervical cancer [2]. Its nuclear expression in cervical cancer cells was positively associated with increased invasiveness, pelvic lymph node metastasis, and late FIGO staging [20]. In ovarian cancer cells, overexpression of Zeb1 induced mesenchymal markers TSHZ1 and FBN1, thus promoting EMT [18]. In endometrial cancer, Zeb1 decreased the expression of E-cadherin [3].

Twist1/2 are basic helix-loop-helix transcription factors sharing 66% structural homology and repress E-cadherin expression in cervical cancer [1, 21]. Twist1 is a master regulator of EMT in cervical cancer and its expression is a poor prognostic factor [1, 22]. Its elevated expression has also been associated with chemo- and radiotherapy resistance in cervical cancers [22, 23]. Twist2 expression in cervical squamous cell carcinoma patients is a predictor for metastatic potential [1, 24]. Expression of these transcription factors in cervical cancer cells is responsible for the activation of AKT and *β*-catenin pathways and for the preservation of stem cell-like characteristics of the cells [1, 25]. Twist overexpression has been demonstrated in invasive endometrial carcinomas and is associated with a lower patient survival rate [3, 26]. It represses the expression of E-cadherin [3]. In ovarian cancer, Twist1 overexpression promotes the expression of N-cadherin and reduces the expression of keratin-8 and E-cadherin [18]. Twist2 plays a role in the induction of EMT in vulvar cancer and has been shown to downregulate E-cadherin [9].

It has been shown that the expression of transcription factor Kruppel-like factor 17 (KLF17) induces the expression of Twist in endometrial cancer cells [27]. This led to the subsequent activation of EMT, increased cell invasion, and drug resistance [27]. Both Twist and KLF17 are upregulated in endometrial cancer cells [27]. KLF4, a transcription factor related to KLF17, acts as a tumor suppressor by inhibiting cell proliferation, migration, and invasion through attenuating transforming growth factor-*β* (TGF-*β*) induced EMT in ovarian cancer cells [28].

Ets variant 5 (ETV5) belongs to the polyoma enhancer activator 3 (PEA3) family of transcription factors and has been found to induce EMT in endometrial cancer cells by increasing the transcription of Zeb1 [14]. Endometrial cancer cell lines that had undergone EMT accompanied by modified cell adhesion molecules and cytoskeleton reorganization were found to have upregulated ETV5 [14, 29]. ETV5 has also been shown to increase the transcription of matrix metalloproteinases 2 (MMP2) and HEP27, which allow for cellular invasion by degrading extracellular matrix and prevent apoptosis in tumor cells, respectively [14, 30, 31].

There is new evidence that E47 and high-mobility group AT-hook 2 (HMGA2) could play a role in inducing EMT in gynecologic cancers. E47 is a basic helix-loop-helix transcription factor that represses the expression of E-cadherin in cervical cancer cells [1]. HMGA2 represses the expression of E-cadherin in endometrial carcinoma cells [3, 32] and is inversely correlated with the expression of tumor suppressor lumican (LUM), an inhibitor of EMT, in ovarian cancer [33].

## 3. Growth Factors

TGF-*β* has been suggested to be associated with the progression of ovarian [34, 35], endometrial [14], and cervical cancers [1]. Overexpression of TGF-*β* (TGF-*β*1, TGF-*β*2, and TGF-*β*3) in ovarian cancer activates a TGF-*β* receptor/Ras-Related C3 botulinum toxin substrate 1 (RAC1)/Smad-dependent signaling pathway [36, 37] and in cervical cancer activates mitogen-activated protein kinases (MAPK), Smad, Wnt, tumor necrosis factor-*α* (TNF-*α*), and nuclear factor-*κ*B (NF-*κ*B) pathways [2, 38], promoting EMT.

Epidermal growth factor (EGF) has been shown to be a potent EMT inducer in a variety of solid tumors, including cervical [39], ovarian [10], and endometrial [14] cancer. In ovarian cancer cells, EGF binds to the EGF receptor (EGFR), thus inducing EMT activation through phosphorylation of the Janus kinase/signal transducer and activator of transcription (JAK/STAT3) pathway. Yagi et al. also showed that heparin-binding epidermal growth factor-like growth factor (HB-EGF) is involved in ovarian cancer metastasis through EMT [40]. Lee et al. have reported that the chronic treatment of EGF in cervical cancer cells increased phosphorylation of GSK-3*β*, a regulator of Snail [41], inducing EMT [39]. The chronic treatment of EGF in cervical cancer cells has been shown to increase phosphorylation of glycogen synthase kinase-3 (GSK-3*β*), a regulator of Snail [41], thus inducing EMT [39]. The suppression of EGF signaling in endometrial cancer cells with EGF inhibitor AG1478 represses EMT and cellular migration and invasion [24].

High levels of hepatocyte growth factor (HGF) and its tyrosine kinase receptor (cMET) are found in malignant ovarian cysts and ascitic fluid from women with ovarian carcinomas [42]. HGF, through activation of the MAPK and phosphatidylinositide-4,5-bisphosphate 3-kinase (PI3K)/AKT pathways, promotes phosphorylation of p70S6K, which induces EMT in ovarian cancer cells by increasing expression of Snail and MMP9 [43, 44].

Vascular endothelial growth factor (VEGF) is an angiogenesis stimulator and its increased expression is associated with tumor growth in endometrial [14] and ovarian cancers [45]. VEGF allows cancers to grow by enabling the development of new blood vessels for nutrient and oxygen supply and metastatic spread. Ptaszynska et al. showed that, in metastatic ovarian cancer cells, VEGF stimulates the expression of protein autotaxin (ATX) [45], a potent motility factor, which produces the bioactive lysophospholipid (LPA) responsible for regulating cellular migration, cell-cell interactions, and inhibiting apoptosis [46]. Furthermore both VEGF and TGF-*β*1 could be responsible for aberrant expression of Ras homolog gene family, member C (RhoC), which is involved in the EMT of ovarian cancer cells [47]. In endometrial cancer, Mirantes et al. showed that VEGF and insulin-like growth factor-1 (IGF1) activate Snail, Slug, Twist, and E47 [3].

Fibroblast growth factor 2 (FGF2), which is a member of the FGF family, induces ovarian cancer cell invasion by the activation of the PI3K/Akt/mammalian target Of rapamycin (mTOR) and MAPK/extracellular signal-regulated kinase (ERK) signaling pathways. This subsequently increases the expression of Slug and ZEB1, which are responsible for E-cadherin downregulation [48].

## 4. Tumor Suppressors

p53 is a well-known tumor suppressor that acts as an inhibitor of EMT [49]. p53 gain-of-function mutants (R273H, R175H, or C135Y) showed downregulation of miR-130b and subsequently increased the expression of Zeb1 and induced EMT in endometrial cancer [50]. Loss-of-function of p53 was also observed in 96% of high-grade serous ovarian cancers [51]. In fact, loss of p53 through Twist activation [52] or its abrogation is associated with mammalian DNA methyltransferase (DNMT) and promoter methylation that represses E-cadherin [53], promoting EMT.

Klotho, a single-pass transmembrane protein, was originally identified as a suppressor of aging [54] and acts as a modulator for insulin [55] and insulin-like growth factor-1 receptor (IGF-1R) [56] signaling and a coreceptor for FGF23 [57]. Mice lacking Klotho have a short lifespan [54] while high levels of Klotho increase lifespan [58]. Studies have demonstrated its role as a tumor suppressor [56, 59, 60]. Klotho has been shown to be downregulated in cervical cancer due to epigenetic silencing of the promoter region [61] and its downregulation is associated with cervical cancer invasiveness [62]. Overexpression of Klotho in cervical cancer cells upregulates E-cadherin and downregulates N-cadherin, Slug, and Twist [62]. In addition, overexpression of Klotho inhibits cell motility and invasiveness by suppressing expression of MMP-7 and MMP-9 and the Wnt/*β*-catenin pathway [62].

Secreted frizzled-related proteins (SFRPs) are extracellular signaling molecules that inhibit the Wnt pathway [63] and are methylated in cervical cancer cells [2, 64]. Overexpression of SFRPs reduces the expression of Twist, Slug, and Snail and upregulates E-cadherin, thus reducing the invasive ability of cervical cancer cells [2, 65]. In ovarian cancer, an* in vitro* study conducted by Su et al. shows that silencing SFRP5 through methylation induces EMT through Twist and activates AKT2 [66].

RAS protein activator-like 2 (RASAL2), a GTPase activating protein (GAP) that has been identified in ovarian cancer as a tumor suppressor, plays an important role in EMT and metastasis [67]. Reduced levels of RASAL2 mRNA activate the Ras-ERK pathway* in vivo* and* in vitro* [67]. Mutations in RAS are very common in malignant diseases and increase the pathological grades of the disease by suppressing E-cadherin and elevating vimentin expression [67], suggesting that downregulation of RASAL2 may promote EMT activation [67].

## 5. Oncogenes

HPV types 16, 18, 33, and 35 have been associated with the development of cervical and vulvar cancers [9]. HPV 16 E6/E7 oncoproteins induce the development of cervical intraepithelial neoplasias [2, 68]. Transfection of normal human foreskin keratinocytes with HPV16 E7 induced the EMT program [1, 69] and cervical cancer cells transfected with HPV16 E6/E7 oncogenes experienced downregulation of E-cadherin and upregulation of vimentin [2, 70]. Lastly, exposure to low-dose estrogen and FGF along with HPV16 E7 transfection induced the production of invasive cervical cancer cells [2, 70].

Src-associated substrate in mitosis of 68 kDa (Sam68) is a member of the signal transducer and activator of RNA (STAR) family of RNA-binding proteins [71] and is involved in various cellular processes, including T cell receptor [72] and insulin receptor signaling [73], transcriptional regulation [74], mRNA processing [75], TNF-induced NF-*κ*B activation, and apoptosis [76]. Li and colleagues have shown that Sam68 is overexpressed in cervical cancer cell lines and tissues and its expression is associated with pelvic lymph node metastasis [77]. Also, its cytoplasmic subcellular localization is associated with poor overall survival of cervical cancer patients [77]. Depletion of Sam68 inhibits migratory and invasive potential of cervical cancer cells as well as the expression of vimentin and fibronectin, possibly through suppressing the Akt/GSK3*β*/Snail pathways [77].

Astrocyte-elevated gene 1 (AEG1) was identified as an HIV-inducible gene in astrocytes [78] and has been implicated in the development, progression, and pathogenesis of various tumors [79–81]. Overexpression of AEG1 resulted in downregulation of E-cadherin and upregulation of N-cadherin and vimentin, thus increasing invasive capability of cervical cancer cells [2, 82]. Also, it is suggested that AEG1 could potentially play a role in the resistance of cervical cancer cells to paclitaxel and cisplatin chemotherapy [2, 82].

Fused-toe homologue (FTS) was originally identified as a gene deleted in the Fused toes mouse mutation [83]. FTS has been involved in uterine cervical carcinogenesis and correlates positively with staging and grading of cervical cancer [2, 84]. Induction of FTS expression reduces expression of E-cadherin and claudin and upregulates expression of N-cadherin and Slug [2, 85]. Knockdown of FTS inhibits EGF-induced EMT and migratory ability of cervical cancer cells [2, 85].

B lymphoma mouse Moloney leukemia virus insertion region 1 (BMI-1) activates EMT in many human cancers and is overexpressed in endometrial cancer [86]. Endometrial cancer cells expressing endogenous BMI-1 show increased invasive ability in comparison to those not expressing endogenous BMI-1 [86]. Those expressing BMI-1 expressed spindle-like, fibroblast morphology, reduced E-cadherin expression, and increased vimentin expression [86]. miR-194 targets BMI-1 and silences its effects, reversing the invasive ability of cells, increasing E-cadherin expression, and decreasing vimentin expression [86]. Furthermore, knockdown of BMI-1 inhibited* in vitro* cell proliferation [86].

## 6. miRNA

miRNAs are small noncoding RNAs that bind target mRNAs and inhibit gene translation [87]. Studies have shown that the miR-200 family, consisting of miR-141, miR-200a, miR-200b, miR-200c, and miR-429, play a crucial role in EMT. miR-200 is a master regulator of cervical cancer and ovarian cancer EMT [2, 51]. Downregulation of miR-200 in mesenchymal cells of uterine carcinosarcomas and ovarian cancer cells increases the expression of Zeb1/2 [3, 51, 88, 89]. A study conducted by Lei et al. has shown that miR-155 functions as a tumor suppressor in cervical cancer cells [90] and other studies have shown its oncogenic role in a variety of human cancer cells and tumors [91–93]. They also reported that overexpression of miR-155 suppresses the migratory/invasive capability of cervical cancer cells and EGF-induced EMT through upregulation of p53 and downregulation of Smad2 [90]. Interestingly, miR-155 was found to be overexpressed in endometrial cancer cells and to induce EMT [14]. It is thought that its mode of action is related to TGF-*β*. When normal murine mammary gland epithelial cells were treated with TGF-*β*, they underwent EMT and also upregulated miR-155 [14, 94]. The ectopic expression of miR-155 led to increased cellular invasiveness and inhibited the formation of tight junctions [14, 94]. Future work should be done to pinpoint the differences in pathways that cause miR-155 to be an EMT suppressor in cervical cancer but an EMT activator in endometrial cancer. miR-361-5p is elevated in cervical cancer cells and promotes cell proliferation, lymph node invasion, and metastasis through EMT [2, 95]. miR-214 [96] and miR-20a [97] promote EMT by downregulation of PTEN in ovarian cancer. Also miR-181a acts as an inducer of TGF-*β* by Smad7 inhibition and promotes EMT in epithelial ovarian cancer [98].

## 7. Other Molecular Factors

Endothelin-1 (ET-1) is a vasoconstrictor peptide that binds two G-protein-coupled transmembrane receptors (ET_A_ and ET_B_) [99, 100] and has been implicated in EMT in ovarian tumors [100]. In particular, elevated levels of ET-1 activate ET_A_ receptor, which activate an integrin-linked kinase- (ILK-) mediated signaling pathway. This causes the inhibition of GSK-3*β* and the E-cadherin promoter and increased levels of *β*-catenin, N-cadherin, and Snail, which drives the cells to a fibroblastoid and invasive phenotype [100]. Also, Colas et al. showed that, in endometrial cancer, ILK signaling activates Snail, Slug, Twist, and E47, promoting EMT [14].

Bone morphogenetic protein 4 (BMP4) belongs to the TGF-*β* superfamily proteins and is highly expressed in ovary cell types [101]. BMP4 in ovarian cancer cells induces EMT via the (BMP4)/anaplastic lymphoma kinase (ALK) pathway, increasing the expression of mRNA and protein levels of Snail and Slug and downregulating E-cadherin [19, 101, 102].

Transmembrane protein receptors, such as integrin, are also involved in cervical [103] and ovarian tumor progression [104]. Integrins consist of 18*α* and 8*β* subunits and they play a major role in cell migration [105]. In cervical cancer, *α*v *β*3 and *α*v *β*6 are associated with decreased patient survival [103]. In ovarian cancer, it has been found that *α*5*β*1 integrin binds to fibronectin to induce EMT [37].

MMPs are a family of zinc-required matrix-degrading enzymes. Several groups have found that the increased expression of MMPs can predict tumor aggressiveness and poor patient survival [106, 107]. A study has shown that cervical cancer cells treated with MMP7 and MMP9 adopt mesenchymal characteristics with high migratory ability [2, 108]. The inhibition of MMP expression causes the inhibition of Vim1, fibronectin, and Snail expression [2, 108]. This suggests that MMPs play a direct role in inducing EMT processes.

Mucins 4/16 (MUC4/16) are identified as tumor antigens in epithelial ovarian cancer. Overexpression of MUC16 in epithelial ovarian cancer downregulates E-cadherin and upregulates N-cadherin and vimentin, thus promoting tumor metastasis [22]. In ovarian cancer cells, MUC4 overexpression upregulates Snail, focal adhesion kinase (FAK), Twist1/2, and MAPK signaling cascade proteins and downregulates E-cadherin and CK18 expression [109].

Chemokine receptor 7 (CCR7) is constitutively expressed in epithelial ovarian cancer. CCR7 and its ligand CCL19/21 participate in EMT development, thus promoting ovarian cancer metastasis [110].

Interleukin 6 (IL6) is a proinflammatory cytokine that induces EMT in cervical and ovarian cancers [2, 111]. In cervical cancer, IL6 promotes EMT activation via the STAT3 pathway [2, 112]. In ovarian cancer, increased levels of EGF stimulate IL6 secretion, which promote the mobility and resistance to chemotherapy via the JAK/STAT3, SHP-2/Ras, MAPK, and PI3K/Akt signaling pathways [111, 113].

Rho-GTPases play a key role in cervical cancer EMT due to its induction of the downregulation of cell adhesion molecules and cytoskeleton reorganization [1]. RhoC is involved in the reorganization of the actin cytoskeleton and in the regulation of cell shape, motility, and attachment [47, 114]. Stable expression of RhoC enhanced migratory, invasive, and tumor-forming abilities of cervical cancer cells [1, 115]. RhoC has been reported to be a downstream effector of Notch receptor [115]. The knockdown of RhoC and Notch receptor caused decreased expression of fibronectin and actin stress fibers during wound healing [1, 115]. Gou et al. showed that overexpression of RhoC downregulates E-cadherin and upregulates B-cadherin and *α*-SMA, thus promoting cell migration, invasion, and lamellipodia formation, and suggested that RhoC-mediated EMT could be initiated by TGF-*β*1 and VEGF [47].

Gelsolin is an actin-binding protein that is upregulated in cervical cancer cells [1, 116]. Cervical cancer patients expressing gelsolin had a decreased 5-year survival rate in comparison to those not expressing gelsolin [1, 116]. Decreased gelsolin expression caused decreased cell migration, MMP2 expression, vimentin, and upregulated E-cadherin expression in cervical cancer cells [1, 116].

Cell surface receptor tyrosine kinase (TrKB) and its ligand, brain-derived neurotrophic factor (BDNF), are upregulated in endometrial carcinoma in comparison to normal epithelial cells [117]. High TrKB levels are associated with EMT phenotype and lymph node metastasis, and its knockdown resulted in the decreased migratory and invasive abilities of the endometrial cancer cells [117].

The expression of transducin *β*-like-related 1 (TBLR1), a transcriptional cofactor, in cervical cancer cells induces the expression of Vim1 and fibronectin while reducing the expression of *β*-catenin [2, 118]. It activates the NF-*κ*B and Wnt/*β*-catenin pathways to upregulate expression of Snail and Twist [2, 118]. Furthermore, TBLR1 expression has been shown to induce the invasiveness of cervical cancer cells [2, 118].

Transforming, acidic coiled-coil protein 3 (TACC3) plays a role in the interaction of actin with microtubules and regulates the centrosome during cell mitosis. We have demonstrated that EGF/EGFR signaling is critical for TACC3-mediated EMT in cervical cancer [119].

K+/Cl− cotransporters (KCC) play an important role in the regulation of cell volume and cytoplasmic chloride, transepithelial transport [120, 121], and cervical tumorigenesis [122, 123]. Hsu et al. have reported that KCC3, one of the KCC isoforms (KCC1–KCC4), is significantly elevated in primary cervical carcinomas and its overexpression causes EMT, accompanied by cell morphological changes, downregulation of E-cadherin and *β*-catenin, upregulation of vimentin, and enhancement of cell proliferation and invasiveness in cervical cancer cells [124].

Erythropoietin-producing human hepatocellular carcinoma (Eph) receptor B2 is a member of the largest family of receptor tyrosine kinases in the human genome [125] and is phosphorylated when its ligand binds, subsequently triggering downstream signaling cascades [126]. Gao et al. have reported that EphB2 is overexpressed in cervical cancer and its expression is associated with tumor progression [127]. In addition, ectopic expression of EphB2 in cervical cancer cells results in an increase in cell migration/invasion and EMT signature fibroblast-like morphology, loss of cell-to-cell contact, and downregulation of E-cadherin. Its ectopic expression also resulted in upregulation of mesenchymal markers (vimentin, CDH2, and fibronectin) and EMT inducers (Snail1, Snail2, and Twist2), as well as an acquisition of stem cell properties via activation of the R-Ras pathway, whereas its depletion has an opposite effect [127].

Nogo isoforms (Nogo-A, Nogo-B, and Nogo-C) belong to the reticulum superfamily and share a conserved reticulum homology domain (RHD), which contains a 66 aa loop domain, Nogo-66 [128]. Nogo-B was identified as a novel human apoptosis-inducing protein [129] and its overexpression has been shown to induce apoptosis through endoplasmic reticulum stress-specific signaling pathways [130] and to play a role in cell adhesion and migration [131, 132]. Nogo-B expression is elevated in cervical cancers and its expression is correlated with the degree of cervical cancer metastasis [128]. Overexpression of Nogo-B in cervical cancer cells promotes cell migration, invasion, and EMT and inhibits cell adhesion [128]. Nogo-B interacts with Fibulin-5, a member of the Fibulin family [133] which contains a conserved Arg-Gly-Asp (RGD) motif that binds to integrins, mediates endothelial cell adhesion, and suppresses angiogenesis [134]. Downregulation of Fibulin-5 inhibits cell migration/invasion, upregulates E-cadherin, and downregulates mesenchymal markers (N-cadherin and vimentin) as well as EMT inducers (Snail, Twist1, Zeb1, and Zeb2) [128].

Estrogen receptors *α* (ER*α*) and *β* (ER*β*) have been recognized in the normal vulvar epithelium [135, 136]. Zannoni et al. showed that changes in both ER*α* and ER*β* expression, likely due to low estrogen, induce EMT in vulvar squamous cell carcinomas, which is associated with a significant reduction in the expression of E-cadherin [136]. In endometrial cancer the reduction of ER*α* correlated with the activation of Wnt, Sonic hedgehog, and TGF-*β* signaling and reduced patient survival [137]. In ovarian cancer, 17*β*-estradiol (E2) promotes EMT via an ER*α*-dependent pathway [138]. In the presence of E2, Snail and Slug are significantly upregulated while E-cadherin is downregulated [138].

Progesterone receptors (PR) have been indicated as potential EMT suppressors in endometrial cancer [14]. PR expression in endometrial cancer is a good prognostic factor for patients and is associated with successful treatment with medroxyprogesterone acetate (MPA) [14, 139]. This may be due to the fact that a loss of progesterone signaling induces EMT. It was shown that MPA treatment in PR-modulated cells downregulates the expression of mesenchymal cell markers, migration, and several signaling pathways, including EGF, IGF1, IL6, integrin/ILK, platelet-derived growth factor (PDGF), TGF-*β*, VEGF, and Wnt/*β*-catenin signaling pathways [14, 140].

Tissue transglutaminase (TG2), a multifunctional protein involved in cellular adhesion, promotes EMT [19] in ovarian carcinoma by activating NF-*κ*B complex, which induces Zeb1 [141]. It also upregulates MMP2 secretion [142] and downregulates E-cadherin [143].

p21-activated kinase (PAK1), a serine/threonine protein kinase, coordinates cell morphology and motility [144] and is targeted by miRNA 222 [145]. PAK1 is highly expressed in primary ovarian cancer and downregulates E-cadherin through Snail [87].

Metastasis-associated gene 1 (MTA1) is considered a critical factor in tumor metastasis [146]. Knockdown of MTA1 decreased migratory, invasive, and adhesive capabilities of cervical cancer cells as well as the expression of E-cadherin and p53 [147]. In ovarian cancer, overexpression of MTA1 promotes oncogenic transformation and downregulates E-cadherin by increasing expression of Snail and Slug [148].

Semaphorin 3E (Sema3E) is a secreted molecule that controls angiogenesis [149] and tumor cell survival and serves as a ligand for Plexin D1 [150]. High levels of Sema3E are found in high-grade ovarian endometrioid carcinoma [151]. Sema3E/Plexin D1 promotes increased migratory and invasive potential and metastatic growth of ovarian endometrioid carcinoma cells [151]. Furthermore, Sema3E/Plexin D1 induces EMT through nuclear localization of Snail and PI3K and ERK/MAPK signaling pathways, which play an important role in the Sema3E/Plexin D1-mediated EMT process in ovarian endometrioid carcinoma cells [151].

GRB2-associated-binding protein 2 (Gab2), a member of the Gab/DOS family of scaffolding adapter proteins [152], is highly expressed in ovarian cancer [153]. Overexpression of Gab2 reduced expression of E-cadherin and increased Zeb1 expression and cell migratory and invasive abilities through the activation of the PI3K pathway in ovarian cancer cells [153].

## 8. Conclusion

Metastasis is the major cause of death from all gynecological cancers. Many studies have found that EMT plays a central role in cancer metastasis by deregulating the molecular network. This allows the downregulation of epithelial markers, upregulation of mesenchymal markers, and increased migration, invasion, cell survival, metastasis, and recurrence.

The knowledge of all factors that contribute to the activation of EMT opens a door to new therapeutic strategies that can inhibit metastasis in all gynecological cancers. Several studies have found that EMT could be pharmacologically targeted. Selumetinib, a small molecule MEK inhibitor, is able to suppress EMT in patients with frequent low-grade serous epithelial ovarian cancer [154]. ILK inhibitors, KP392, QLT0267, and T315, can suppress ILK- EMT activity in epithelial ovarian cancer [155].* In vitro* studies have also shown that the EGF inhibitor, AG1478, causes EGF-induced EMT in cervical, ovarian, and endometrial cancer [155]. Additionally, it has been found that inhibitors of FAK (focal adhesion kinase, induced by MUC4-overexpressing cells) [109], ETaR (zibotentan), and TG2 (KCC-009) suppress EMT in ovarian cancer [155]. Furthermore, vaccines and antibodies against mucins (MUC16) can limit tumor metastasis in ovarian carcinoma [113]. New improvements have been made in targeting EMT via epigenetic or miRNA control in epithelial ovarian cancer. For example, restoration of the miR-200c family negatively regulates the EMT process [154]. HPV vaccine targeting HPV16 E6/E7 antigens in patients with high-grade cervical dysplasias actually caused regression of the dysplasias [156]. Lapatinib, a drug used in endometrial cancers, blocks EGFR tyrosine kinase, thereby preventing EGF signaling [157]. The rapid understanding of molecular dysregulation involved in EMT activation creates the hope for future approaches to more personalized treatments that will have a positive impact on survival rate.

## Figures and Tables

**Table 1:** Factors involved in EMT of gynecological cancers.

	Cervical cancer	Ovarian cancer	Endometrial cancer	Vulvar cancer	Vaginal cancer
Transcriptional regulators	Snail/Slug/Smuc Zeb1/Zeb2 Twist1/Twist2 E47	Snail Zeb1/2 Twist1 KLF4 ETV5 HMGA2	Snail/Slug Zeb1 Twist1/Twist2 KLF17 ETV5 HMGA2	Snail/Slug Twist2	

Growth factors	TGF-*β* EGF	TGF-*β* EGF /HB-EGF VEGF HGF FGF	TGF-*β* EGF VEGF IGF1		

Oncogenes	HPV16 E6/E7 Sam68 AEG1 FTS	PIK3CA AKT2	BMI-1		

Tumor suppressors	Klotho SFRPs	P53 SFRPs RASAL2	P53		

miRNAs	miR-200 family miR-155 miR-361-5p	miR-200 family miR-181 miR-20a miR-214	miR-200 family miR-155 miR-130		

Other molecular factors	*β*1-integrin receptors MMPs 7/9 IL6 RhoC Gelsolin TBLR1 TACC3 KCC3 EphB2 Nogo-B TNF-*α*	*β*1-integrin receptors MMP 9 IL6 RhoC MUC4/16 ET-1 BMP4 CCR7 TG2 PAK1 MTA1 Sema3E Gb2 FN1	ER*α* TrkB PR	ER*α*/*β*	

## References

[1] Lee M.-Y., Shen M.-R. (2012). Epithelial-mesenchymal transition in cervical carcinoma. *American Journal of Translational Research*.

[2] Qureshi R., Arora H., Rizvi M. A. (2015). EMT in cervical cancer: its role in tumour progression and response to therapy. *Cancer Letters*.

[3] Mirantes C., Espinosa I., Ferrer I., Dolcet X., Prat J., Matias-Guiu X. (2013). Epithelial-to-mesenchymal transition and stem cells in endometrial cancer. *Human Pathology*.

[4] Siegel R., Ma J., Zou Z., Jemal A. (2014). Cancer statistics, 2014. *Cancer Journal for Clinicians*.

[5] Agarwal R., Kaye S. B. (2003). Ovarian cancer: strategies for overcoming resistance to chemotherapy. *Nature Reviews Cancer*.

[6] Coleman R. L., Monk B. J., Sood A. K., Herzog T. J. (2013). Latest research and treatment of advanced-stage epithelial ovarian cancer. *Nature Reviews Clinical Oncology*.

[7] Leitao M. M., Chi D. S. (2009). Surgical management of recurrent ovarian cancer. *Seminars in Oncology*.

[8] Sturgeon S. R., Brinton L. A., Devesa S. S., Kurman R. J. (1992). In situ and invasive vulvar cancer incidence trends (1973 to 1987). *The American Journal of Obstetrics and Gynecology*.

[9] Rodrigues I. S., Lavorato-Rocha A. M., Maia B. D. M. (2013). Epithelial-mesenchymal transition-like events in vulvar cancer and its relation with HPV. *British Journal of Cancer*.

[10] Yue P., Zhang X., Paladino D. (2012). Hyperactive EGF receptor, Jaks and Stat3 signaling promote enhanced colony-forming ability, motility and migration of cisplatin-resistant ovarian cancer cells. *Oncogene*.

[11] Wintzell M., Löfstedt L., Johansson J., Pedersen A. B., Fuxe J., Shoshan M. (2012). Repeated cisplatin treatment can lead to a multiresistant tumor cell population with stem cell features and sensitivity to 3-bromopyruvate. *Cancer Biology & Therapy*.

[12] Haslehurst A. M., Koti M., Dharsee M. (2012). EMT transcription factors snail and slug directly contribute to cisplatin resistance in ovarian cancer. *BMC Cancer*.

[13] Marchini S., Fruscio R., Clivio L. (2013). Resistance to platinum-based chemotherapy is associated with epithelial to mesenchymal transition in epithelial ovarian cancer. *European Journal of Cancer*.

[14] Colas E., Pedrola N., Devis L. (2012). The EMT signaling pathways in endometrial carcinoma. *Clinical and Translational Oncology*.

[15] Zhao W., Zhou Y., Xu H., Cheng Y., Kong B. H. (2013). Snail family proteins in cervical squamous carcinoma: expression and significance. *Clinical and Investigative Medicine*.

[16] Ikenouchi J., Matsuda M., Furuse M., Tsukita S. (2003). Regulation of tight junctions during the epithelium-mesenchyme transition: direct repression of the gene expression of claudins/occludin by Snail. *Journal of Cell Science*.

[17] Kao Y.-C., Wu L.-W., Shi C.-S. (2010). Downregulation of thrombomodulin, a novel target of snail, induces tumorigenesis through epithelial-mesenchymal transition. *Molecular and Cellular Biology*.

[18] Davidowitz R. A., Selfors L. M., Iwanicki M. P. (2014). Mesenchymal gene program-expressing ovarian cancer spheroids exhibit enhanced mesothelial clearance. *The Journal of Clinical Investigation*.

[19] Davidson B., Tropé C. G., Reich R. (2012). Epithelial-mesenchymal transition in ovarian carcinoma. *Frontiers in Oncology*.

[20] Chen Z., Li S., Huang K. (2013). The nuclear protein expression levels of SNAI1 and ZEB1 are involved in the progression and lymph node metastasis of cervical cancer via the epithelial-mesenchymal transition pathway. *Human Pathology*.

[21] Franco H. L., Casasnovas J., Rodríguez-Medina J. R., Cadilla C. L. (2011). Redundant or separate entities?—roles of Twist1 and Twist2 as molecular switches during gene transcription. *Nucleic Acids Research*.

[22] Shibata K., Kajiyama H., Ino K. (2008). Twist expression in patients with cervical cancer is associated with poor disease outcome. *Annals of Oncology*.

[23] Zhu K., Chen L., Han X., Wang J. (2012). Short hairpin RNA targeting Twist1 suppresses cell proliferation and improves chemosensitivity to cisplatin in HeLa human cervical cancer cells. *Oncology Reports*.

[24] Li Y., Wang W., Wang W. (2012). Correlation of TWIST2 up-regulation and epithelial-mesenchymal transition during tumorigenesis and progression of cervical carcinoma. *Gynecologic Oncology*.

[25] Li J., Zhou B. P. (2011). Activation of beta-catenin and Akt pathways by Twist are critical for the maintenance of EMT associated cancer stem cell-like characters. *BMC Cancer*.

[26] Kyo S., Sakaguchi J., Ohno S. (2006). High twist expression is involved in infiltrative endometrial cancer and affects patient survival. *Human Pathology*.

[27] Dong P., Kaneuchi M., Xiong Y. (2014). Identification of KLF17 as a novel epithelial to mesenchymal transition inducer via direct activation of TWIST1 in endometrioid endometrial cancer. *Carcinogenesis*.

[28] Chen Z., Wang Y., Liu W. (2014). Doxycycline inducible Kruppel-like factor 4 lentiviral vector mediates mesenchymal to epithelial transition in ovarian cancer cells. *PLoS ONE*.

[29] Colas E., Muinelo-Romay L., Alonso-Alconada L. (2012). ETV5 cooperates with LPP as a sensor of extracellular signals and promotes EMT in endometrial carcinomas. *Oncogene*.

[30] Monge M., Colas E., Doll A. (2007). ERM/ETV5 up-regulation plays a role during myometrial infiltration through matrix metalloproteinase-2 activation in endometrial cancer. *Cancer Research*.

[31] Monge M., Colas E., Doll A. (2009). Proteomic approach to ETV5 during endometrial carcinoma invasion reveals a link to oxidative stress. *Carcinogenesis*.

[32] Montserrat N., Mozos A., Llobet D. (2012). Epithelial to mesenchymal transition in early stage endometrioid endometrial carcinoma. *Human Pathology*.

[33] Wu J., Liu Z., Shao C. (2011). HMGA2 overexpression-induced ovarian surface epithelial transformation is mediated through regulation of EMT genes. *Cancer Research*.

[34] Do T.-V., Kubba L. A., Du H., Sturgis C. D., Woodruff T. K. (2008). Transforming growth factor-*β*1, transforming growth factor-*β*2, and transforming growth factor-*β*3 enhance ovarian cancer metastatic potential by inducing a Smad3-dependent epithelial-to-mesenchymal transition. *Molecular Cancer Research*.

[35] Xu Z., Jiang Y., Steed H., Davidge S., Fu Y. (2010). TGF*β* and EGF synergistically induce a more invasive phenotype of epithelial ovarian cancer cells. *Biochemical and Biophysical Research Communications*.

[36] Bartlett J. M., Langdon S. P., Scott W. N. (1997). Transforming growth factor-*β* isoform expression in human ovarian tumours. *European Journal of Cancer*.

[37] Kenny H. A., Chiang C.-Y., White E. A. (2014). Mesothelial cells promote early ovarian cancer metastasis through fibronectin secretion. *Journal of Clinical Investigation*.

[38] Kloth J. N., Fleuren G. J., Oosting J. (2005). Substantial changes in gene expression of Wnt, MAPK and TNF*α* pathways induced by TGF-*β*1 in cervical cancer cell lines. *Carcinogenesis*.

[39] Lee M.-Y., Chou C.-Y., Tang M.-J., Shen M.-R. (2008). Epithelial-mesenchymal transition in cervical cancer: correlation with tumor progression, epidermal growth factor receptor overexpression, and snail up-regulation. *Clinical Cancer Research*.

[40] Yagi H., Yotsumoto F., Miyamoto S. (2008). Heparin-binding epidermal growth factor-like growth factor promotes transcoelomic metastasis in ovarian cancer through epithelial-mesenchymal transition. *Molecular Cancer Therapeutics*.

[41] Zhou B. P., Deng J., Xia W. (2004). Dual regulation of Snail by GSK-3*β*-mediated phosphorylation in control of epithelial-mesenchymal transition. *Nature Cell Biology*.

[42] Sowter H. M., Corps A. N., Smith S. K. (1999). Hepatocyte growth factor (HGF) in ovarian epithelial tumour fluids stimulates the migration of ovarian carcinoma cells. *International Journal of Cancer*.

[43] Zhou H. Y., Wong A. S. T. (2006). Activation of p70S6K induces expression of matrix metalloproteinase 9 associated with hepatocyte growth factor-mediated invasion in human ovarian cancer cells. *Endocrinology*.

[44] Pon Y. L., Zhou H. Y., Cheung A. N. Y., Ngan H. Y. S., Wong A. S. T. (2008). p70 S6 kinase promotes epithelial to mesenchymal transition through snail induction in ovarian cancer cells. *Cancer Research*.

[45] Ptaszynska M. M., Pendrak M. L., Bandle R. W., Stracke M. L., Roberts D. D. (2008). Positive feedback between vascular endothelial growth factor-A and autotaxin in ovarian cancer cells. *Molecular Cancer Research*.

[46] Gil O. D., Lee C., Ariztia E. V. (2008). Lysophosphatidic acid (LPA) promotes E-cadherin ectodomain shedding and OVCA429 cell invasion in an uPA-dependent manner. *Gynecologic Oncology*.

[47] Gou W.-F., Zhao Y., Lu H. (2014). The role of RhoC in epithelial-to-mesenchymal transition of ovarian carcinoma cells. *BMC Cancer*.

[48] Lau M.-T., So W.-K., Leung P. C. K. (2013). Fibroblast growth factor 2 induces E-cadherin down-regulation via PI3K/Akt/mTOR and MAPK/ERK signaling in ovarian cancer cells. *PLoS ONE*.

[49] Chang C. J., Chao C. H., Xia W. (2011). p53 regulates epithelial-mesenchymal transition and stem cell properties through modulating miRNAs. *Nature Cell Biology*.

[50] Dong P., Karaayvaz M., Jia N. (2013). Mutant p53 gain-of-function induces epithelial-mesenchymal transition through modulation of the miR-130b-ZEB1 axis. *Oncogene*.

[51] Kwon M. J., Shin Y. K. (2011). Epigenetic regulation of cancer-associated genes in ovarian cancer. *International Journal of Molecular Sciences*.

[52] Yang J., Mani S. A., Donaher J. L. (2004). Twist, a master regulator of morphogenesis, plays an essential role in tumor metastasis. *Cell*.

[53] Cheng J.-C., Auersperg N., Leung P. C. K. (2011). Inhibition of p53 represses e-cadherin expression by increasing DNA methyltransferase-1 and promoter methylation in serous borderline ovarian tumor cells. *Oncogene*.

[54] Kuro-o M., Matsumura Y., Aizawa H. (1997). Mutation of the mouse *klotho* gene leads to a syndrome resembling ageing. *Nature*.

[55] Utsugi T., Ohno T., Ohyama Y. (2000). Decreased insulin production and increased insulin sensitivity in the klotho mutant mouse, a novel animal model for human aging. *Metabolism*.

[56] Wolf I., Levanon-Cohen S., Bose S. (2008). Klotho: a tumor suppressor and a modulator of the IGF-1 and FGF pathways in human breast cancer. *Oncogene*.

[57] Urakawa I., Yamazaki Y., Shimada T. (2006). Klotho converts canonical FGF receptor into a specific receptor for FGF23. *Nature*.

[58] Kurosu H., Yamamoto M., Clark J. D. (2005). Physiology: suppression of aging in mice by the hormone Klotho. *Science*.

[59] Abramovitz L., Rubinek T., Ligumsky H. (2011). KL1 internal repeat mediates klotho tumor suppressor activities and inhibits bFGF and IGF-I signaling in pancreatic cancer. *Clinical Cancer Research*.

[60] Zhu Y., Xu L., Zhang J. (2013). Klotho suppresses tumor progression via inhibiting PI3K/Akt/GSK3*β*/Snail signaling in renal cell carcinoma. *Cancer Science*.

[61] Lee J., Jeong D.-J., Kim J. (2010). The anti-aging gene *KLOTHO* is a novel target for epigenetic silencing in human cervical carcinoma. *Molecular Cancer*.

[62] Chang B., Kim J., Jeong D. (2012). Klotho inhibits the capacity of cell migration and invasion in cervical cancer. *Oncology Reports*.

[63] Hiramoto T., Oka T., Yoshihara K., Kubo C. (2009). Pyrogenic cytokines did not mediate a stress interview-induced hyperthermic response in a patient with psychogenic fever. *Psychosomatic Medicine*.

[64] Chung M. T., Sytwu H. K., Yan M. D. (2009). Promoter methylation of SFRPs gene family in cervical cancer. *Gynecologic Oncology*.

[65] Chung M.-T., Lai H.-C., Sytwu H.-K. (2009). SFRP1 and SFRP2 suppress the transformation and invasion abilities of cervical cancer cells through Wnt signal pathway. *Gynecologic Oncology*.

[66] Su H.-Y., Lai H.-C., Lin Y.-W. (2010). Epigenetic silencing of SFRP5 is related to malignant phenotype and chemoresistance of ovarian cancer through Wnt signaling pathway. *International Journal of Cancer*.

[67] Huang Y., Zhao M., Xu H. (2014). RASAL2 down-regulation in ovarian cancer promotes epithelial-mesenchymal transition and metastasis. *Oncotarget*.

[68] Schiffman M., Wentzensen N., Wacholder S., Kinney W., Gage J. C., Castle P. E. (2011). Human papillomavirus testing in the prevention of cervical cancer. *Journal of the National Cancer Institute*.

[69] Hellner K., Mar J., Fang F., Quackenbush J., Münger K. (2009). HPV16 E7 oncogene expression in normal human epithelial cells causes molecular changes indicative of an epithelial to mesenchymal transition. *Virology*.

[70] Cheng Y.-M., Chou C.-Y., Hsu Y.-C., Chen M.-J., Wing L. Y. C. (2012). The role of human papillomavirus type 16 E6/E7 oncoproteins in cervical epithelial-mesenchymal transition and carcinogenesis. *Oncology Letters*.

[71] Lukong K. E., Richard S. (2003). Sam68, the KH domain-containing superSTAR. *Biochimica et Biophysica Acta (BBA)—Reviews on Cancer*.

[72] Fusaki N., Iwamatsu A., Iwashima M., Fujisawa J.-I. (1997). Interaction between Sam68 and Src family tyrosine kinases, Fyn and Lck, in T cell receptor signaling. *The Journal of Biological Chemistry*.

[73] Najib S., Martín-Romero C., González-Yanes C., Sánchez-Margalet V. (2005). Role of Sam68 as an adaptor protein in signal transduction. *Cellular and Molecular Life Sciences*.

[74] Sánchez-Jiménez F., Sánchez-Margalet V. (2013). Role of Sam68 in post-transcriptional gene regulation. *International Journal of Molecular Sciences*.

[75] Paronetto M. P., Messina V., Bianchi E. (2009). Sam68 regulates translation of target mRNAs in male germ cells, necessary for mouse spermatogenesis. *Journal of Cell Biology*.

[76] Ramakrishnan P., Baltimore D. (2011). Sam68 is required for both NF-*κ*B activation and apoptosis signaling by the TNF receptor. *Molecular Cell*.

[77] Li Z., Yu C.-P., Zhong Y. (2012). Sam68 expression and cytoplasmic localization is correlated with lymph node metastasis as well as prognosis in patients with early-stage cervical cancer. *Annals of Oncology*.

[78] Kang D. C., Su Z. Z., Sarkar D., Emdad L., Volsky D. J., Fisher P. B. (2005). Cloning and characterization of HIV-1-inducible astrocyte elevated gene-1, AEG-1. *Gene*.

[79] Yoo B. K., Emdad L., Su Z.-Z. (2009). Astrocyte elevated gene-1 regulates hepatocellular carcinoma development and progression. *The Journal of Clinical Investigation*.

[80] Long M., Dong K., Gao P. (2013). Overexpression of astrocyte-elevated gene-1 is associated with cervical carcinoma progression and angiogenesis. *Oncology Reports*.

[81] Yu C., Chen K., Zheng H. (2009). Overexpression of astrocyte elevated gene-1 (AEG-1) is associated with esophageal squamous cell carcinoma (ESCC) progression and pathogenesis. *Carcinogenesis*.

[82] Liu X., Wang D., Liu H. (2014). Knockdown of astrocyte elevated gene-1 (AEG-1) in cervical cancer cells decreases their invasiveness, epithelial to mesenchymal transition, and chemoresistance. *Cell Cycle*.

[83] Lesche R., Peetz A., Van Der Hoeven F., Rüther U. (1997). *Ftl*, a novel gene related to ubiquitin-conjugating enzymes, is deleted in the Fused toes mouse mutation. *Mammalian Genome*.

[84] Cinghu S., Anandharaj A., Lee H.-C., Yu J.-R., Park W.-Y. (2011). FTS (fused toes homolog) a novel oncoprotein involved in uterine cervical carcinogenesis and a potential diagnostic marker for cervical cancer. *Journal of Cellular Physiology*.

[85] Muthusami S., Prabakaran D. S., Yu J.-R., Park W.-Y. (2014). EGF-induced expression of Fused Toes Homolog (FTS) facilitates epithelial-mesenchymal transition and promotes cell migration in ME180 cervical cancer cells. *Cancer Letters*.

[86] Dong P., Kaneuchi M., Watari H. (2011). MicroRNA-194 inhibits epithelial to mesenchymal transition of endometrial cancer cells by targeting oncogene BMI-1. *Molecular Cancer*.

[87] Vaksman O., Stavnes H. T., Kærn J., Trope C. G., Davidson B., Reich R. (2011). miRNA profiling along tumour progression in ovarian carcinoma. *Journal of Cellular and Molecular Medicine*.

[88] Iorio M. V., Visone R., di Leva G. (2007). MicroRNA signatures in human ovarian cancer. *Cancer Research*.

[89] Nam E. J., Yun M. J., Oh Y. T. (2010). Diagnosis and staging of primary ovarian cancer: correlation between PET/CT, Doppler US, and CT or MRI. *Gynecologic Oncology*.

[90] Lei C., Wang Y., Huang Y., Yu H., Wu L., Huang L. (2012). Up-regulated miR155 reverses the epithelial-mesenchymal transition induced by EGF and increases chemo-sensitivity to cisplatin in human Caski cervical cancer cells. *PLoS ONE*.

[91] Zhang C. M., Zhao J., Deng H. Y. (2013). MiR-155 promotes proliferation of human breast cancer MCF-7 cells through targeting tumor protein 53-induced nuclear protein 1. *Journal of Biomedical Science*.

[92] Zhao X.-D., Zhang W., Liang H.-J., Ji W.-Y. (2013). Overexpression of miR -155 promotes proliferation and invasion of human laryngeal squamous cell carcinoma via targeting SOCS1 and STAT3. *PLoS ONE*.

[93] Ryu J. K., Hong S.-M., Karikari C. A., Hruban R. H., Goggins M. G., Maitra A. (2010). Aberrant microRNA-155 expression is an early event in the multistep progression of pancreatic adenocarcinoma. *Pancreatology*.

[94] Kong W., Yang H., He L. (2008). MicroRNA-155 is regulated by the transforming growth factor *β*/Smad pathway and contributes to epithelial cell plasticity by targeting RhoA. *Molecular and Cellular Biology*.

[95] Wu X., Xi X., Yan Q. (2013). MicroRNA-361-5p facilitates cervical cancer progression through mediation of epithelial-to-mesenchymal transition. *Medical Oncology*.

[96] Yang H., Kong W., He L. (2008). MicroRNA expression profiling in human ovarian cancer: miR-214 induces cell survival and cisplatin resistance by targeting PTEN. *Cancer Research*.

[97] Wang Y., Kim S., Kim I. M. (2014). Regulation of metastasis by microRNAs in ovarian cancer. *Frontiers in Oncology*.

[98] Parikh A., Lee C., Joseph P. (2014). MicroRNA-181a has a critical role in ovarian cancer progression through the regulation of the epithelial-mesenchymal transition. *Nature Communications*.

[99] Martin E. R., Brenner B. M., Ballermann B. J. (1990). Heterogeneity of cell surface endothelin receptors. *The Journal of Biological Chemistry*.

[100] Rosanò L., Spinella F., di Castro V. (2005). Endothelin-1 promotes epithelial-to-mesenchymal transition in human ovarian cancer cells. *Cancer Research*.

[101] Vergara D., Merlot B., Lucot J.-P. (2010). Epithelial-mesenchymal transition in ovarian cancer. *Cancer Letters*.

[102] Thériault B. L., Shepherd T. G., Mujoomdar M. L., Nachtigal M. W. (2007). BMP4 induces EMT and Rho GTPase activation in human ovarian cancer cells. *Carcinogenesis*.

[103] Desgrosellier J. S., Cheresh D. A. (2010). Integrins in cancer: biological implications and therapeutic opportunities. *Nature Reviews Cancer*.

[104] Casey R. C., Burleson K. M., Skubitz K. M. (2001). *β*1-integrins regulate the formation and adhesion of ovarian carcinoma multicellular spheroids. *The American Journal of Pathology*.

[105] Brakebusch C., Bouvard D., Stanchi F., Sakai T., Fässler R. (2002). Integrins in invasive growth. *The Journal of Clinical Investigation*.

[106] Jones L. E., Humphreys M. J., Campbell F., Neoptolemos J. P., Boyd M. T. (2004). Comprehensive analysis of matrix metalloproteinase and tissue inhibitor expression in pancreatic cancer: increased expression of matrix metalloproteinase-7 predicts poor survival. *Clinical Cancer Research*.

[107] Katayama A., Bandoh N., Kishibe K. (2004). Expressions of matrix metalloproteinases in early-stage oral squamous cell carcinoma as predictive indicators for tumor metastases and prognosis. *Clinical Cancer Research*.

[108] Lin C.-Y., Tsai P.-H., Kandaswami C. C. (2011). Matrix metalloproteinase-9 cooperates with transcription factor Snail to induce epithelial-mesenchymal transition. *Cancer Science*.

[109] Ponnusamy M. P., Lakshmanan I., Jain M. (2010). MUC4 mucin-induced epithelial to mesenchymal transition: a novel mechanism for metastasis of human ovarian cancer cells. *Oncogene*.

[110] Cheng S., Han L., Guo J., Yang Q., Zhou J., Yang X. (2014). The essential roles of CCR7 in epithelial-to-mesenchymal transition induced by hypoxia in epithelial ovarian carcinomas. *Tumor Biology*.

[111] Touboul C., Lis R., Al Farsi H. (2013). Mesenchymal stem cells enhance ovarian cancer cell infiltration through IL6 secretion in an amniochorionic membrane based 3D model. *Journal of Translational Medicine*.

[112] Miao J.-W., Liu L.-J., Huang J. (2014). Interleukin-6-induced epithelial-mesenchymal transition through signal transducer and activator of transcription 3 in human cervical carcinoma. *International Journal of Oncology*.

[113] Hiss D. (2012). Optimizing molecular-targeted therapies in ovarian cancer: the renewed surge of interest in ovarian cancer biomarkers and cell signaling pathways. *Journal of Oncology*.

[114] Parri M., Chiarugi P. (2010). Rac and Rho GTPases in cancer cell motility control. *Cell Communication and Signaling*.

[115] Srivastava S., Ramdass B., Nagarajan S., Rehman M., Mukherjee G., Krishna S. (2010). Notch1 regulates the functional contribution of RhoC to cervical carcinoma progression. *British Journal of Cancer*.

[116] Liao C. J., Wu T. I., Huang Y. H. (2011). Overexpression of gelsolin in human cervical carcinoma and its clinicopathological significance. *Gynecologic Oncology*.

[117] Bao W., Qiu H., Yang T., Luo X., Zhang H., Wan X. (2013). Upregulation of TrkB promotes epithelial-mesenchymal transition and anoikis resistance in endometrial carcinoma. *PLoS ONE*.

[118] Wang J., Ou J., Guo Y. (2014). TBLR1 is a novel prognostic marker and promotes epithelial-mesenchymal transition in cervical cancer. *British Journal of Cancer*.

[119] Ha G. H., Kim J. L., Breuer E. K. Y. (2013). TACC3 is essential for EGF-mediated EMT in cervical cancer. *PLoS ONE*.

[120] Boettger T., Rust M. B., Maier H. (2003). Loss of K-Cl co-transporter KCC3 causes deafness, neurodegeneration and reduced seizure threshold. *The EMBO Journal*.

[121] Adragna N. C., Di Fulvio M., Lauf P. K. (2004). Regulation of K-Cl cotransport: from function to genes. *The Journal of Membrane Biology*.

[122] Shen M.-R., Chou C.-Y., Ellory J. C. (2000). Volume-sensitive KCl cotransport associated with human cervical carcinogenesis. *Pflügers Archiv*.

[123] Shen M.-R., Chou C.-Y., Hsu K.-F. (2003). KCl cotransport is an important modulator of human cervical cancer growth and invasion. *Journal of Biological Chemistry*.

[124] Hsu Y.-M., Chen Y.-F., Chou C.-Y. (2007). KCl cotransporter-3 down-regulates E-cadherin/*β*-catenin complex to promote epithelial-mesenchymal transition. *Cancer Research*.

[125] Nakada M., Hayashi Y., Hamada J.-I. (2011). Role of Eph/ephrin tyrosine kinase in malignant glioma. *Neuro-Oncology*.

[126] Kullander K., Klein R. (2002). Mechanisms and functions of Eph and ephrin signalling. *Nature Reviews Molecular Cell Biology*.

[127] Gao Q., Liu W., Cai J. (2014). EphB2 promotes cervical cancer progression by inducing epithelial- mesenchymal transition. *Human Pathology*.

[128] Xiao W., Zhou S., Xu H. (2013). Nogo-B promotes the epithelial-mesenchymal transition in HeLa cervical cancer cells via Fibulin-5. *Oncology Reports*.

[129] Qi B., Qi Y., Watari A. (2003). Pro-apoptotic ASY/Nogo-B protein associates with ASYIP. *Journal of Cellular Physiology*.

[130] Kuang E., Wan Q., Li X., Xu H., Zou T., Qi Y. (2006). ER stress triggers apoptosis induced by Nogo-B/ASY overexpression. *Experimental Cell Research*.

[131] Su Z., Cao L., Zhu Y. (2007). Nogo enhances the adhesion of olfactory ensheathing cells and inhibits their migration. *Journal of Cell Science*.

[132] Liao H., Duka T., Teng F. Y. H. (2004). Nogo-66 and myelin-associated glycoprotein (MAG) inhibit the adhesion and migration of Nogo-66 receptor expressing human glioma cells. *Journal of Neurochemistry*.

[133] Albig A. R., Schiemann W. P. (2005). Fibulin-5 function during tumorigenesis. *Future Oncology*.

[134] Xie L., Palmsten K., MacDonald B. (2008). Basement membrane derived fibulin-1 and fibulin-5 function as angiogenesis inhibitors and suppress tumor growth. *Experimental Biology and Medicine*.

[135] Schwartz P. E. (2000). The oestrogen receptor (ER) in vulva, vagina and ovary. *European Journal of Cancer*.

[136] Zannoni G. F., Prisco M. G., Vellone V. G., de Stefano I., Scambia G., Gallo D. (2011). Changes in the expression of oestrogen receptors and E-cadherin as molecular markers of progression from normal epithelium to invasive cancer in elderly patients with vulvar squamous cell carcinoma. *Histopathology*.

[137] Wik E., Ræder M. B., Krakstad C. (2013). Lack of estrogen receptor-*α* is associated with epithelial-mesenchymal transition and PI3K alterations in endometrial carcinoma. *Clinical Cancer Research*.

[138] Park S.-H., Cheung L. W. T., Wong A. S. T., Leung P. C. K. (2008). Estrogen regulates snail and slug in the down-regulation of E-cadherin and induces metastatic potential of ovarian cancer cells through estrogen receptor *α*. *Molecular Endocrinology*.

[139] Thigpen J. T., Brady M. F., Alvarez R. D. (1999). Oral medroxyprogesterone acetate in the treatment of advanced or recurrent endometrial carcinoma: a dose-response study by the gynecologic oncology group. *Journal of Clinical Oncology*.

[140] van der Horst P. H., Wang Y., Vandenput I. (2012). Progesterone inhibits epithelial-to-mesenchymal transition in endometrial cancer. *PLoS ONE*.

[141] Cao L., Shao M., Schilder J., Guise T., Mohammad K. S., Matei D. (2012). Tissue transglutaminase links TGF-beta, epithelial to mesenchymal transition and a stem cell phenotype in ovarian cancer. *Oncogene*.

[142] Satpathy M., Shao M., Emerson R., Donner D. B., Matei D. (2009). Tissue transglutaminase regulates matrix metalloproteinase-2 in ovarian cancer by modulating cAMP-response element-binding protein activity. *The Journal of Biological Chemistry*.

[143] Shao M., Cao L., Shen C. (2009). Epithelial-to-mesenchymal transition and ovarian tumor progression induced by tissue transglutaminase. *Cancer Research*.

[144] Sells M. A., Boyd J. T., Chernoff J. (1999). p21-Activated kinase 1 (Pak1) regulates cell motility in mammalian fibroblasts. *The Journal of Cell Biology*.

[145] Zhao H., Yao R., Cao X., Wu G. (2011). Neuroimmune modulation following traumatic stress in rats: evidence for an immunoregulatory cascade mediated by c-Src, miRNA222 and PAK1. *Journal of Neuroinflammation*.

[146] Toh Y., Nicolson G. L. (2009). The role of the MTA family and their encoded proteins in human cancers: molecular functions and clinical implications. *Clinical and Experimental Metastasis*.

[147] Rao Y., Wang H., Fan L., Chen G. (2011). Silencing MTA1 by RNAi reverses adhesion, migration and invasiveness of cervical cancer cells (SiHa) via altered expression of p53, and E-cadherin/beta-catenin complex. *Journal of Huazhong University of Science and Technology—Medical Science*.

[148] Dannenmann C., Shabani N., Friese K., Jeschke U., Mylonas I., Brüning A. (2008). The metastasis-associated gene MTA1 is upregulated in advanced ovarian cancer, represses ER*β*, and enhances expression of oncogenic cytokine GRO. *Cancer Biology and Therapy*.

[149] Tamagnone L., Rehman M. (2013). To die or not to die: sema3E rules the game. *Cancer Cell*.

[150] Luchino J., Hocine M., Amoureux M.-C. (2013). Semaphorin 3E suppresses tumor cell death triggered by the plexin D1 dependence receptor in metastatic breast cancers. *Cancer Cell*.

[151] Tseng C.-H., Murray K. D., Jou M.-F., Hsu S.-M., Cheng H.-J., Huang P.-H. (2011). Sema3E/plexin-D1 mediated epithelial-to-mesenchymal transition in ovarian endometrioid cancer. *PLoS ONE*.

[152] Gu H., Neel B. G. (2003). The ‘Gab’ in signal transduction. *Trends in Cell Biology*.

[153] Wang Y., Sheng Q., Spillman M. A., Behbakht K., Gu H. (2012). Gab2 regulates the migratory behaviors and E-cadherin expression via activation of the PI3K pathway in ovarian cancer cells. *Oncogene*.

[154] Huang R. Y.-J., Chung V. Y., Thiery J. P. (2012). Targeting pathways contributing to epithelial-mesenchymal transition (EMT) in epithelial ovarian cancer. *Current Drug Targets*.

[155] Davis F. M., Stewart T. A., Thompson E. W., Monteith G. R. (2014). Targeting EMT in cancer: opportunities for pharmacological intervention. *Trends in Pharmacological Sciences*.

[156] Maldonado L., Teague J. E., Morrow M. P. (2014). Intramuscular therapeutic vaccination targeting HPV16 induces T cell responses that localize in mucosal lesions. *Science Translational Medicine*.

[157] Konecny G. E., Venkatesan N., Yang G. (2008). Activity of lapatinib a novel HER2 and EGFR dual kinase inhibitor in human endometrial cancer cells. *British Journal of Cancer*.

